# Whole exome sequencing in thrombophilic pedigrees to identify genetic risk factors for venous thromboembolism

**DOI:** 10.1371/journal.pone.0187699

**Published:** 2017-11-08

**Authors:** Marisa L. R. Cunha, Joost C. M. Meijers, Frits R. Rosendaal, Astrid van Hylckama Vlieg, Pieter H. Reitsma, Saskia Middeldorp

**Affiliations:** 1 Department of Experimental Vascular Medicine, Academic Medical Center, University of Amsterdam, Amsterdam, the Netherlands; 2 Department of Vascular Medicine, Academic Medical Center, University of Amsterdam, Amsterdam, the Netherlands; 3 Department of Plasma Proteins, Sanquin, Amsterdam, the Netherlands; 4 Department of Clinical Epidemiology, Leiden University Medical Center, Leiden, the Netherlands; 5 Department of Thrombosis and Hemostasis, Leiden University Medical Center, Leiden, the Netherlands; 6 Einthoven Laboratory for Experimental Vascular Medicine, Leiden University Medical Center, Leiden, the Netherlands; Institut d'Investigacions Biomediques de Barcelona, SPAIN

## Abstract

**Background:**

Family studies have shown a strong heritability component for venous thromboembolism (VTE), but established genetic risk factors are present in only half of VTE patients.

**Aim:**

To identify genetic risk factors in two large families with unexplained hereditary VTE.

**Methods:**

We performed whole exome sequencing in 10 affected relatives of two unrelated families with an unexplained tendency for VTE. We prioritized variants shared by all affected relatives from both families, and evaluated these in the remaining affected and unaffected individuals. We prioritized variants based on 3 different filter strategies: variants within candidate genes, rare variants across the exome, and SNPs present in patients with familial VTE and with low frequency in the general population. We used whole exome sequencing data available from 96 unrelated VTE cases with a positive family history of VTE from an affected sib study (the GIFT study) to identify additional carriers and compared the risk-allele frequencies with the general population. Variants found in only one individual were also retained for further analysis. Finally, we assessed the association of these variants with VTE in a population-based case-control study (the MEGA study) with 4,291 cases and 4,866 controls.

**Results:**

Six variants remained as putative disease-risk candidates. These variants are located in 6 genes spread among 3 different loci: 2p21 (*PLEKHH2* NM_172069:c.3105T>C, *LRPPRC* rs372371276, *SRBD1* rs34959371), 5q35.2 (*UNC5A* NM_133369.2:c.1869+23C>A), and 17q25.1 (*GPRC5C* rs142232982, *RAB37* rs556450784). In GIFT, additional carriers were identified only for the variants located in the 2p21 locus. In MEGA, additional carriers for several of these variants were identified in both cases and controls, without a difference in prevalence; no carrier of the *UNC5A* variant was present.

**Conclusion:**

Despite sequencing of several individuals from two thrombophilic families resulting in 6 candidate variants, we were unable to confirm their relevance as novel thrombophilic defects.

## Background

Venous thromboembolism (VTE), comprising deep vein thrombosis (DVT) and pulmonary embolism (PE), is a common disorder with a high mortality rate worldwide [1]. After a first episode of VTE patients have an elevated risk of a recurrent episode that is as high as 30% to 50% within 10 years in those with unprovoked VTE [2]. Major acquired risk factors for VTE include cancer, trauma, surgery, immobilization, pregnancy, use of oral contraceptives and the antiphospholipid syndrome. A family history of VTE increases the risk as well and indicates an important underlying genetic component [3–5].

Sequencing-based studies and, to a lesser extent, genome-wide association studies (GWAs), have contributed to the discovery of novel genetic risk factors for VTE [6–14] Rare mutations identified by sequencing of candidate genes, i.e. antithrombin, protein C and protein S, are those that convey the highest risk [15]. Yet, the currently known genetic risk factors are present in approximately half of the patients with unexplained but familial VTE [16], which suggests that other genetic risk factors remain to be identified. The discovery of such genetic causes of VTE may unravel novel disease-causing mechanisms in or outside the coagulation cascade.

The aim of this study was to identify novel genetic risk factors for VTE by whole exome sequencing of 10 individuals from two unrelated Dutch families with an unexplained tendency for VTE, to estimate the prevalence of any candidate variants among patients with familial VTE, and to subsequently validate these in a large population-based case control study. We hypothesized that unexplained familial VTE is caused by the presence of genetic variants located in coding regions of genes with known or unknown roles in hemostasis.

## Materials and methods

### Subjects

**Fig 1:**
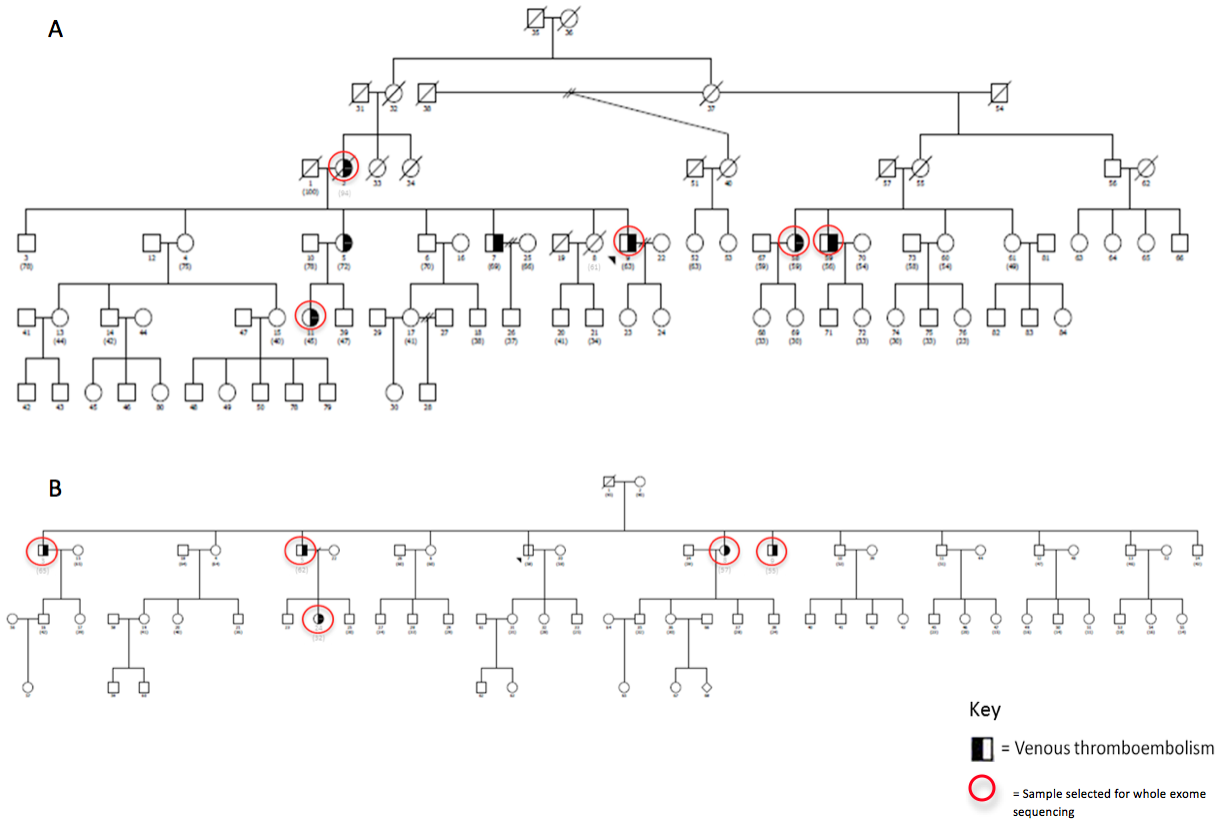
Pedigree of the families with inherited VTE. Half shaded symbols indicate affected individuals. In family D (A), 7 individuals had had objectively confirmed VTE while in family K (B), 5 individuals had had objectively confirmed VTE. Exome data was generated from all individuals surrounded by a red circle. Please notice that although the affected individual 5 from family D was not genetically tested, the genotype of her daughter (individual 11) who also has the same phenotype, is available. Therefore, it can be assumed that all candidate mutations validated in individual 11 are also present in the individual 5. Moreover, none of the individuals from the oldest generation of family K had had an objectively confirmed VTE event. The VTE events for each of the affected individuals from family D are the following: DVT in the leg at age of 46 for individual 59; DVT in the leg at age of 48 for individual 58; DVT in the leg and in the arm, and PE at age of 42, 50, and 52, respectively, for individual 9; DVT in the leg at age of 26 for individual 2; PE at age of 22 for individual 11; DVT in the leg at age of 30 for individual 5, and; DVT in the leg at age of 56 for individual 7. The VTE events for each of the affected individuals from family K are the following: PE at age of 44 for individual 9; DVT in the abdomen at age of 41, 42 and 45 for individual 8; DVT in the leg at age of 53 for individual 5; PE at the age of 43 and 45 for individual 3 and; PE at age of 23 for individual 24.

Two large Dutch families, referred to as Family D and Family K, were selected from the GENES study, which has been described previously [17]. In both families, 5 or more individuals had had objectively confirmed VTE, in the absence of known hereditary thrombophilia (antithrombin-, protein C-, and protein S deficiency, factor V Leiden and prothrombin G20210A). All affected individuals from both families had experienced VTE at a relatively young age and some individuals had had DVT at unusual sites (abdomen or arms). The pedigrees are depicted in Fig 1.

In addition to the two families, two sets of patients with familial VTE, as well as a population-based case-control study of VTE were included in this study. The patients with familial VTE comprised of 36 unrelated cases with familial VTE from the GENES study [17], and 434 cases with VTE from 201 families from the GIFT study, which included affected sibs with thrombosis before the age of 45, in the absence of known thrombophilia [18]. The population-based MEGA study consisted of 4,291 cases with a first VTE and 4,866 controls [19]. Identification of cases and controls has been described in detail previously [17–19]. The GENES study was approved by the Medical Ethics Committee of the Academic Medical Center, Amsterdam, the Netherlands. The GIFT and the MEGA studies were approved by the Medical Ethics Committee of the Leiden University Medical Center, Leiden, the Netherlands. Written informed consent was obtained from all participants.

### Whole-exome sequencing

#### Families

Genomic DNA extracted from whole blood from 5 affected relatives from both families was sent to the Beijing Genomics Institute for whole exome sequencing Fig 1. The exomes were captured using the Agilent SureSelect Human all Exon (44M) kit and the enriched exome libraries were multiplexed and sequenced on the Illumina HiSeq2000 platform to generate 90-bp paired-end reads per individual with an average sequencing depth above 50x. The exome design covers 44 Mb of human genome corresponding to the exons and flanking intronic regions of ~18,000 genes in the National Center for Biotechnology Information Consensus CDS database (April 2011 release).

The sequencing reads were aligned to the human reference genome sequence NCBI Build 37 (hg19) using the Burrows-Wheeler Aligner (BWA) version (v) 0.6.2. Then, each SAM file resulting from each sample was converted to a BAM file using SAMtools (v0.1.18). Picard tools (v1.78) and GATK tools (v2.2) were used to process the BAM files. Variant calling was performed with all samples simultaneously. Single nucleotide variants/polymorphisms (here globally called as SNPs), as well as insertions and deletions (INDELs), were called by the UnifiedGenotyper walker from GATK tools. After quality score recalibration and removal of low-confidence variants, all SNPs and INDELs were annotated using ANNOVAR (November 2012 release). All steps were performed according to GATK Best Practices recommendations [20].

#### GIFT study

Genomic DNA extracted from whole blood from 96 unrelated cases with familial VTE from the GIFT study was sent to the The Post-Genomic Platform of the Pitié-Salpêtrière (P3S) for whole exome sequencing. The DNA libraries were performed using TruSeq DNA sample Prep kit (Illumina) and the exomes were captured using the TruSeq Exome Enrichment kit (Illumina). The enriched exome libraries were multiplexed and sequenced on the Illumina HiSeq2000 platform to generate 100-bp paired-end reads per individual with an average sequencing depth above 50x. The exome design covers 62 Mb of human genome corresponding to the exons and UTR regions of 20794 genes.

Illumina's CASAVA software v1.8.2 was used to demultiplex samples and convert BCL files to FASTQ format files. Quality controls of the raw data were performed with FastQC (v0.10.1). Reads with poor quality were removed (fastq_illumina_filter-0.1) and low quality bases of the reads were trimmed (sickle v1.200). After these preprocessing steps, sequenced data were aligned to the human genome reference sequence NCBI Build 37 (hg19) using BWA (v0.6.2). Reads in the resulting BAM files were then realigned around INDELs and the base quality score were recalibrated using GATK (v2.4–3). Duplicates and singletons were then removed along with non-well oriented reads using Picard (v1.85) and Samtools (v0.1.18). Variant calling was performed on bases with good quality (Q-score>20) using the samtools mpileup command. Variants that were present in GIFT and in individuals from families D and K were identified.

### Filter strategies

**Fig 2:**
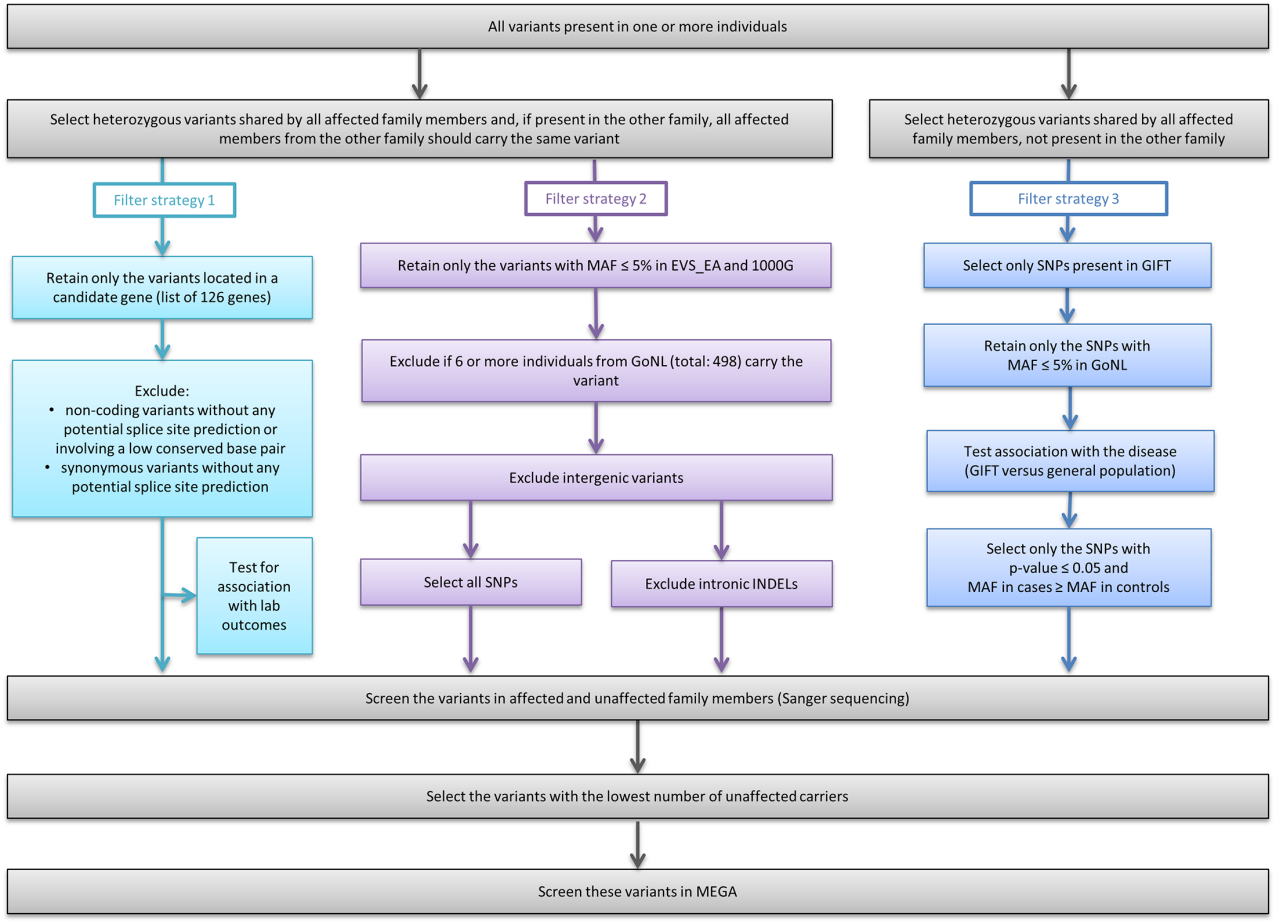
Overview of all filter strategies applied to the whole exome sequencing data derived from the DNA of 10 affected individuals from 2 Dutch families with inherited VTE (5 from each family). Three filter strategies were applied to the sequencing data. These strategies were based on: variants within candidate genes (filter strategy 1), rare variants across the exome (filter strategy 2) and, SNPs present in patients with familial VTE and rare in the general population, i.e., associated with VTE (filter strategy 3). Together, all these strategies might increase the chances of finding the genetic risk factor for VTE present in family D and family K. Abbreviations: SNPs, single nucleotide variants/polymorphisms; INDELs, insertions and deletions; MAF, minor allele frequency; GoNL, Genome of the Netherlands project database, 498 unrelated Dutch individuals; EVS_EA, NHLBI Exome Sequencing Project database, 4,300 European-American unrelated individuals; 1000G, 1000 Genomes Project, 2,500 individuals from about 25 populations around the world; GIFT, Genetics In Familial Thrombosis study, 96 unrelated VTE cases with positive family history of VTE; MEGA, Multiple Environmental and Genetic Assessment of risk factors for venous thrombosis study, up to 4,291 cases with VTE and 4,866 controls.

To identify the genetic variant responsible for the increased risk for VTE in members from the families D and K, we applied 3 different strategies, summarized in Fig 2. These strategies were based on: (i) variants within candidate genes (filter strategy 1), (ii) rare variants across the exome (filter strategy 2), (iii) SNPs present in patients with familial VTE and with low frequency (minor allele frequency < 5%) in the general population (filter strategy 3). All strategies assume that familial VTE is an autosomal dominant disease, and that all affected family members from a certain family share the same genetic risk variant. In addition, variants located in the X chromosome were considered for analysis in filter strategies 1 and 2, as VTE affects both males and females.

For filter strategy 1, we created a list of genes known or possibly associated with VTE based on the available literature. This list comprises a total of 126 genes and is available in S1 File. Following the selection of SNPs and INDELs located within these candidate genes, we enriched for variants more likely to affect protein function, by excluding non-coding variants without any potential splice site prediction or involving a low conserved base pair, as well as synonymous variants without any potential splice site prediction. Then, we tested the effect of these variants on hemostatic traits in cases from the GIFT study, although we realize that this approach is subject to collider bias [21,22]. We analysed 413 cases, for which previously investigated hemostatic traits and genotypes were available from a previous genome-wide association study [14]. For more information on the association analysis see further below in this section.

For filter strategy 2, we identified all variants with minor allele frequency (MAF) equal or less than 5% in the NHLBI Exome Sequencing Project database, 4,300 European-American unrelated individuals (EVS_EA) [23] and 1000G database [24]. Then, we selected only variants identified in 5 or less (≤1%) individuals from GoNL, as these individuals share the same ethnic origin and geographic region as families D and K. To minimize genotyping errors, all intergenic variants and INDELs located exclusively in intronic regions were excluded from further analysis.

For filter strategy 3, we identified SNPs that were present in one or more GIFT cases for whom whole exome sequencing data was available, and with MAF equal or below than 5% in GoNL. We tested these SNPs for association with VTE by comparing the risk allele frequencies in GIFT (n = 96) with those present in GoNL (n = 498) or EVS_EA databases (n = 4,300). SNPs shared by members from both families were not considered in this filter strategy. Of note, SNPs available only in the GIFT database, i.e., only identified in cases with VTE, are retained for follow-up analysis in filter strategy 2.

After applying all the filter strategies, we evaluated each of the retained variants to minimize the presence of sequencing errors (e.g. those inherent to the technology used) [25,26]. Variants located in duplicated genome regions, in homopolymeric regions or with MAF above 10% in 1000G or EVS_EA databases (resulting from filter strategy 3) were excluded from further analysis. Furthermore, when 2 or more SNPs were located in the same gene, we used SNiPA [27] to evaluate whether these SNPs were in high linkage disequilibrium (D'>0.9), and if so only one was taken for further analysis.

Finally, variants not present in dbSNP135 were classified as novel variants in this study. Yet, to facilitate the identification of all variants mentioned in this text, we have annotated these variants with a later version of dbSNP database, the dbSNP142.

### Confirmation with Sanger sequencing or allele-specific PCR

DNA samples available from affected and unaffected members from both families were used for validation and segregation analysis of the candidate variants. This was performed by standard bidirectional Sanger sequencing. We ranked the variants in terms of number of asymptomatic carriers within a family, and we used samples from the GENES and the MEGA studies for follow-up validation of the top ranked variants (i.e., those with the lowest number of asymptomatic carriers). In total, 6 variants were selected for follow-up validation: 2 variants from family D and 4 variants from family K. These variants were validated with Sanger sequencing or allele-specific PCR on DNA available from 36 samples from the GENES study and 9,157 samples from the MEGA study, respectively (primers available on request).

### Statistical analysis

#### Testing for association between a SNP and VTE

For each SNP resulting from filter strategy 3, the allele frequencies between cases (GIFT) and controls (GoNL or EVS_EA) were compared using the Pearson’s chi-square test with 1 degree of freedom. Associations with p<0.05 were defined as putative associations. After applying Bonferroni correction, associations with p<0.0003 (0.05/135) were considered to be statistically significant. SNPs located in the X chromosome were excluded from this analysis, since sex information from the individuals enrolled in GoNL was not available. Instead, we decided to select for further analysis all coding SNPs or novel non-coding SNPs located in the X chromosome, which were absent in GoNL database. The association between each SNP genotyped in MEGA and VTE was investigated using Fisher’s exact test. The threshold for significance was set as p< 0.05. The statistical analysis was performed using the software R (version 2.15.2).

#### Association of SNPs from candidate genes on the variability of hemostatic traits

We performed linear regression analyses adjusted for age and sex to estimate the effect of each SNP resulted from filter strategy 1 on hemostatic traits known to associate with VTE. Investigated hemostatic traits were endogenous thrombin generation, plasma antigen or activity levels of fibrinogen, coagulation factors II, VII, VIII, IX, X, and XII, von Willebrand factor, antithrombin, protein C, protein S (total and free), protein Z, C4b binding protein, and anti-β2-glycoprotein I. The threshold for significance was set as p< 0.05 and when we corrected for multiple testing, p< 0.0004 was considered to be statistically significant. We repeated this analysis using some SNPs resulted from filter strategy 2 but solely for 3 hemostatic traits: fibrinogen, protein S total and protein S free levels. The statistical analysis was performed using the software SPSS (version 22).

### Sample size considerations

Using standard power calculation methods for case-control studies, the MEGA study would be sufficient to detect relative risks of 4 for a dominant trait with a minor allele frequency of 0.1%, with a power of 80% and 5% two-sided type I-error[28].

## Results

### Whole exome sequencing

#### Families

The sequencing yielded an average sequencing depth of ~53x per sample, with at least 86% of the targeted region covered more than 10x. Each individual had ~64,000 SNPs and ~12,000 INDELs, making in total 121,347 SNPs and 23,364 INDELs to undergo our filtering strategies (Fig 2).

#### GIFT study

Sequence data was successfully generated for all 96 subjects. In the combined 96 samples, the average read depth was 63X and the median was 62X. This value ranged from 38X to 80X according to individuals. In 91 samples, the average is above 50X, in the remaining 5 samples, the average read depth was around 40X. For all samples, the coverage was over 10x at greater than 88% of the targeted region. The median [range] number of SNPs and INDELs with read depth above 10x detected per sample was 87,461 [78,956–90,627] and 2,768 [2,249–2,997], respectively.

Overall, 13,286 genes carried at least one new or rare (<0.5%) coding variant (based on reported 1000g2012apr_eur frequency), and the average number of such variants per individual was 1,456.

### Variants shared by all affected relatives

We identified all single nucleotide polymorphisms and insertions and deletions shared by all affected relatives from the families D and K. Tables 1 and 2 list the variants identified in autosomes and X chromosome, respectively.

**Table 1:** Number of variants located in autosomes retained after each filter step. Abbreviations: SNPs, single nucleotide variants/polymorphisms; INDELs, insertions and deletions; MAF, minor allele frequency; GoNL, Genome of the Netherlands project database, 498 unrelated Dutch individuals; EA, NHLBI Exome Sequencing Project database, 4,300 European-American unrelated individuals; 1000G, 1000 Genomes Project, 2,500 individuals from about 25 populations around the world; VTE, venous thromboembolism; GIFT, Genetics In Familial Thrombosis study, 96 unrelated VTE cases with positive family history of VTE.

		SNPs			INDELs		
Filter strategy	Filter step	Exclusive to family D	Exclusive to family K	Shared by both families	Exclusive to family D	Exclusive to family K	Shared by both families
**1,2,3**	1. Present in all affected individuals	995	1467	21029	80	89	2073
	2. Only heterozygous	276	733	207	22	40	101
							
**1**	3. Within candidate genes	4	7	0	0	0	0
	4. Retain only with functional effect	2	3	0	0	0	0
	**Selected for Sanger sequencing**	**2**	**3**	**0**	**0**	**0**	**0**
							
**2**	3. MAF_EA < = 5%	111	391	140	14	28	92
	4. MAF_1000G < = 5%	36	201	90	12	20	86
	5. Less than 6 carriers in GoNL (total of 498 unrelated controls)	10	56	10	12	20	83
	6. Exclude intergenic	10	54	2	10	20	69
	7. INDELs: Exclude intronic	10	54	2	3	3	4
	**Selected for Sanger sequencing**	**10**	**48**	**0**	**2**	**2**	**1**
							
**3**	3. Present in GIFT (total of 96 index cases)	265	663	-	-	-	-
	4. MAF_GoNL < = 5%	28	167	-	-	-	-
	5. Retain putative associations with VTE (p-value< = 0.05)	6	13	-	-	-	-
	6. Remove if MAF in controls > MAF in cases	5	10	-	-	-	-
	**Selected for Sanger sequencing**	**3**	**6**	**-**	**-**	**-**	**-**
							
**1,2,3**	**Total variants for Sanger sequencing**	**14**	**53**	**0**	**2**	**2**	**1**

**Table 2:** Number of variants located in the X chromosome retained after each filter step. Abbreviations: SNPs, single nucleotide variants/polymorphisms; INDELs, insertions and deletions; MAF, minor allele frequency; GoNL, Genome of the Netherlands project database, 498 unrelated Dutch individuals; EA, NHLBI Exome Sequencing Project database, 4,300 European-American unrelated individuals; 1000G, 1000 Genomes Project, 2,500 individuals from about 25 populations around the world.

		SNPs			INDELs		
Filter strategy	Filter step	Exclusive to family D	Exclusive to family K	Shared by both families	Exclusive to family D	Exclusive to family K	Shared by both families
**1,2**	1. Present in all affected individuals	8	30	339	0	0	45
	2. Only heterozygous females and homozygous males	2	20	0	0	0	0
							
**1**	3. Within candidate genes	0	0	0	0	0	0
	4. Retain only with functional effect	0	0	0	0	0	0
	**Selected for Sanger sequencing**	**0**	**0**	**0**	**0**	**0**	**0**
							
**2**	3. MAF_EA < = 5%	1	15	0	0	0	0
	4. MAF_1000G< = 5%	0	6	0	0	0	0
	5. Less than 6 carriers in GoNL	0	1	0	0	0	0
	6. Exclude intergenic	0	1	0	0	0	0
	7. INDELs: Exclude intronic	0	1	0	0	0	0
	**Selected for Sanger sequencing**	**0**	**1**	**0**	**0**	**0**	**0**
							
**1,2**	**Total variants for Sanger sequencing**	**0**	**1**	**0**	**0**	**0**	**0**

#### Autosomal variants

We identified 276 SNPs and 22 INDELs exclusively shared by all affected individuals from family D, 733 SNPs and 40 INDELs exclusively shared by all affected individuals from family K, and 207 SNPs and 101 INDELs shared by all affected individuals from both families. All these variants were present in a heterozygous form. Only few SNPs were novel: 3 SNPs exclusive to family D, 36 SNPs exclusive to family K and 9 SNPs shared by both families.

#### Variants located in the X chromosome

We identified 2 and 20 SNPs, but no INDELs, shared by all affected individuals from family D and family K, respectively. Only 1 SNP was novel. The 10 individuals with these 22 SNPs shared no SNPs.

### Selection of the candidate variants

Tables 1 and 2 display the number of variants retained after each filtering step. Concerning the variants exclusive to family D, 2 SNPs, 10 SNPs and 3 INDELs, and 5 SNPs remained after applying all the filtering steps from the filter strategies 1, 2, and 3. One SNP, *MAP3K6* rs55841735, was common between 2 of the 3 filter strategies. Concerning the variants exclusive to family K, 3 SNPs, 55 SNPs and 3 INDELs, and 10 SNPs remained after applying all the filtering steps from the filter strategies 1, 2, and 3. Four SNPs, *LRPPRC* rs372371276, *SRBD1* rs34959371, *FSCN3* rs34941808, and *ZNF816* rs369724877 were common between 2 of the 3 filter strategies. Regarding the variants shared by all 10 affected individuals, no variants remained for analysis, except for filter strategy 2. Using this filter strategy, 2 SNPs and 4 INDELs remained for analysis. Of note, 1 of the variants that resulted from filter strategy 2 was identified only among cases of VTE (family K and GIFT). This was an intronic SNP in the *NEBL* gene (rs61849814).

After all the variants have been individually checked, we excluded 12 SNPs and 5 INDELs from further validation with Sanger sequencing. Five of these variants were intronic SNPs from family K, which we considered not relevant for further analysis (details can be found in S2 File).

The 73 candidate variants retained for validation (68 SNPs and 5 INDELs) are listed in the Table 3 along with the predicted impact on protein function, and MAF in various databases.

**Table 3:** List of the 73 variants selected for validation. Abbreviations: Chr, Chromosome; Ref, reference allele; Obs, observed allele; HOM, homozygous; HET, heterozygous; Func, variant function; SNPs, single nucleotide variants/polymorphisms; MAF, minor allele frequency; GoNL, Genome of the Netherlands project database, 498 unrelated Dutch individuals; EVS_EA, NHLBI Exome Sequencing Project database, 4,300 European-American unrelated individuals; 1000G, 1000 Genomes Project, 2,500 individuals from about 25 populations around the world; GIFT, Genetics In Familial Thrombosis study, 96 unrelated VTE cases with positive family history of VTE; GoNL, Genome of the Netherlands project database, 498 unrelated Dutch individuals.

Chr	Start	End	Ref	Obs	Func	Gene	Exonic Func	dbSNP135	MAF EA_6500_%	MAF GIFT_% (assuming_allCalled)	MAF 1000G 2012apr_ALL_%	GoNL number of genotypes (HOM_Ref/HET/HOM_Obs)	Filter strategy
1	1905479	1905479	C	T	exonic	*KIAA1751*	nonsynonymous SNP		NA	0.00	0	NA	2
1	6211042	6211042	C	G	intronic	*CHD5*			0.05	0.00	0	NA	2
1	27688743	27688743	T	C	splicing	*MAP3K6*		rs55841735	0.41	1.56	0.41	494/4/0	2.3
1	117122285	117122285	-	TCC	exonic	*IGSF3*	nonframeshift insertion		NA	21.77	0	NA	2
1	156354348	156354348	C	-	splicing	*RHBG*		rs11303415	NA	0.00	0	NA	2
1	231377078	231377078	C	A	UTR5	*GNPAT*			0.01	0.00	0	493/1/0	2
1	234536943	234536943	T	G	exonic	*TARBP1*	nonsynonymous SNP		NA	0.00	0	NA	2
2	43965641	43965641	T	C	exonic	*PLEKHH2*	synonymous SNP		NA	0.52	0	NA	2
2	44184604	44184604	T	C	intronic	*LRPPRC*			0.02	0.52	0	NA	2.3
2	45640302	45640302	G	C	exonic	*SRBD1*	nonsynonymous SNP	rs34959371	0.43	1.04	0.27	497/1/0	2.3
2	207175476	207175476	G	T	exonic	*ZDBF2*	nonsynonymous SNP	rs36095066	0.87	0.00	0.46	493/5/0	2
3	42251580	42251580	-	GGA	exonic	*TRAK1*	nonframeshift insertion		NA	0.00	0	NA	2
3	107932728	107932728	G	A	intronic	*IFT57*			0.40	0.52	0.09	496/2/0	2
3	111667982	111667982	C	T	intronic	*PHLDB2*		rs116705395	NA	0.00	0.18	497/1/0	2
3	121097497	121097497	C	T	intronic	*STXBP5L*		rs182808568	NA	0.00	0.14	494/4/0	2
3	121260373	121260373	G	A	intronic	*POLQ*		rs183249693	0.08	0.00	0.09	494/4/0	2
3	124829057	124829057	A	G	exonic	*SLC12A8*	synonymous SNP	rs11714448	3.09	6.25	1	470/28/0	3
3	129308276	129308276	G	A	exonic	*PLXND1*	nonsynonymous SNP	rs146072632	0.21	0.00	0	481/1/0	2
3	195510838	195510838	G	A	exonic	*MUC4*	nonsynonymous SNP		NA	0.00	0	NA	2
3	195591020	195591020	T	G	UTR3	*TNK2*		rs112362099	1.00	2.60	1	480/18/0	3
4	144346515	144346515	C	G	intronic	*GAB1*		rs187533266	NA	0.00	0.23	497/1/0	2
5	176305151	176305151	C	A	intronic	*UNC5A*			0.01	0.00	0	NA	2
5	180661310	180661310	G	T	exonic	*TRIM41*	synonymous SNP		0.03	0.00	0	NA	2
7	100016781	100016781	T	C	exonic	*ZCWPW1*	nonsynonymous SNP	rs141450215	0.50	0.00	0.27	496/2/0	2
7	103061356	103061356	T	A	intronic	*SLC26A5*			NA	0.00	0	497/1/0	2
7	110763503	110763503	A	G	exonic	*LRRN3*	synonymous SNP	rs35229264	0.47	0.00	0.09	495/3/0	2
7	111936315	111936315	C	T	exonic	*ZNF277*	synonymous SNP	rs142208106	0.00	0.00	0	496/2/0	2
7	127229265	127229265	C	T	intronic	*ARF5*			0.56	1.56	0.23	487/4/0	2
7	127239585	127239585	C	T	exonic	*FSCN3*	nonsynonymous SNP	rs34941808	0.60	1.56	0.14	495/3/0	2.3
9	2039776	2039776	-	CAG	exonic	*SMARCA2*	nonframeshift insertion		NA	11.54	0	NA	2
9	2648286	2648286	G	A	exonic	*VLDLR*	nonsynonymous SNP	rs35339834	0.13	0.00	0.05	497/1/0	2
9	5919929	5919929	T	C	exonic	*KIAA2026*	nonsynonymous SNP		0.07	0.00	0	NA	2
9	8486132	8486132	G	C	exonic	*PTPRD*	synonymous SNP	rs144111555	0.10	0.00	0.27	496/2/0	2
9	136917482	136917482	G	A	exonic	*BRD3*	synonymous SNP	rs61731642	0.00	0.00	0.09	NA	2
9	140145895	140145895	C	G	ncRNA_intronic	*LOC100129722*			0.17	0.52	0	NA	2
10	21318672	21318672	A	C	intronic	*NEBL*		rs61849814	NA	1.04	0	NA	2
11	62601915	62601915	G	A	exonic	*WDR74*	nonsynonymous SNP		NA	0.00	0	NA	2
11	63883847	63883847	C	T	exonic	*FLRT1*	synonymous SNP		NA	0.00	0	NA	2
11	65293664	65293664	G	A	exonic	*SCYL1*	nonsynonymous SNP		0.01	0.00	0	NA	2
11	66472221	66472221	G	A	exonic	*SPTBN2*	synonymous SNP	rs144939155	0.25	0.52	0.18	479/3/0	2
11	66638474	66638474	C	A	intronic	*PC*			NA	0.00	0	NA	2
11	68177405	68177405	C	T	exonic	*LRP5*	synonymous SNP	rs145456776	0.03	0.00	0	NA	2
12	130919182	130919182	G	A	intronic	*RIMBP2*			NA	0.00	0	NA	2
12	131311749	131311749	A	C	exonic	*STX2*	nonsynonymous SNP	rs137928907	2.55	2.08	1	463/32/1	1
12	133306808	133306808	C	T	exonic	*ANKLE2*	nonsynonymous SNP	rs149597645	1.63	3.65	1	477/21/0	3
13	113824899	113824899	T	C	intronic	*PROZ*		rs3024787	NA	0.00	0	493/5/0	2
14	105360291	105360291	C	G	intronic	*KIAA0284*		rs185909273	NA	0.00	0.18	496/2/0	2
14	105418571	105418571	C	T	exonic	*AHNAK2*	nonsynonymous SNP		0.00	0.00	0	NA	2
16	602505	602505	C	T	exonic	*SOLH*	synonymous SNP		0.00	0.00	0	NA	2
16	4940366	4940366	A	G	intronic	*PPL*			0.14	0.52	0.18	497/1/0	2
16	5083391	5083391	C	G	exonic	*NAGPA*	nonsynonymous SNP		NA	0.00	0	NA	2
17	45360730	45360730	T	C	exonic	*ITGB3*	nonsynonymous SNP	rs5918	15.17	14.58	9	341/145/12	1
17	64210757	64210757	C	A	exonic	*APOH*	nonsynonymous SNP	rs4581	23.76	21.88	47	311/166/21	1
17	65713943	65713943	-	A	upstream	*NOL11*			NA	0.00	0	NA	2
17	66274384	66274384	C	T	exonic	*SLC16A6*	synonymous SNP	rs149995088	0.29	0.52	0.09	494/4/0	2
17	67310584	67310584	A	G	UTR5	*ABCA5*			NA	0.00	0	NA	2
17	72443156	72443156	G	A	exonic	*GPRC5C*	nonsynonymous SNP	rs142232982	0.05	0.00	0.09	NA	2
17	72740355	72740355	G	A	intronic	*RAB37*			NA	0.00	0	NA	2
18	43496563	43496563	G	A	intronic	*EPG5*			0.12	0.00	0	497/1/0	2
19	15569378	15569378	G	C	exonic	*RASAL3*	nonsynonymous SNP	rs58123634	4.84	6.77	3	457/25/0	3
19	47207685	47207685	G	C	intronic	*PRKD2*			0.08	0.00	0	494/1/0	2
19	49254538	49254538	T	G	exonic	*FUT1*	nonsynonymous SNP		NA	0.00	0	NA	2
19	49713534	49713534	G	A	exonic	*TRPM4*	nonsynonymous SNP		NA	0.00	0	NA	2
19	49878275	49878275	G	A	exonic	*DKKL1*	nonsynonymous SNP	rs35389403	0.33	0.00	1	NA	2
19	49910139	49910139	C	G	intronic	*CCDC155*		rs112074780	0.39	0.00	1	NA	2
19	50163098	50163098	G	A	intronic	*IRF3*			NA	0.00	0	NA	2
19	51503285	51503285	C	T	exonic	*KLK8*	nonsynonymous SNP	rs16988799	5.15	2.60	5	441/56/1	1
19	51528041	51528041	C	T	exonic	*KLK11*	nonsynonymous SNP	rs3745539	7.53	7.29	6	421/71/5	1
19	53454862	53454862	T	C	intronic	*ZNF816*			0.08	0.52	0	497/1/0	2.3
20	744270	744270	G	A	exonic	*SLC52A3*	synonymous SNP	rs139430185	0.05	0.00	0	497/1/0	2
22	38890624	38890624	A	C	intronic	*DDX17*			NA	0.00	0	497/1/0	2
22	41736075	41736075	C	A	exonic	*ZC3H7B*	synonymous SNP		NA	0.00	0	494/2/0	2
X	117702143	117702143	C	G	intronic	*DOCK11*			0.07		0	NA	2

### Validation of candidate variants

We designed primers to validate the 73 candidate variants resulting from all filter strategies.

The presence of 5 variants, 4 SNPs (*IFT57* rs199895727, *IRF3* NM_001197122.1:c.1115-8C>T, *MUC4* rs199718845 and *TRIM41* rs376725370) and 1 INDEL (*TRAK1* NM_001265609:c.1844_1845insGGA) could not be validated due to PCR problems. The presence of the remaining variants was evaluated by Sanger sequencing in affected and unaffected family members (including 1 additional affected member from family D for whom no whole exome sequencing data were available) (S3 File). We identified the variants with the lowest number of unaffected carriers based on the families’ genotyping results. We selected 6 variants. These variants were located in 3 different loci: 2p21 (*PLEKHH2*, *LRPPRC* and *SRBD1*) and 5q35.2 (*UNC5A*) based on family K, and 17q25.1 (*GPRC5C* and *RAB37*) based on family D. Three of these variants, all from the 2p21 locus, were also present in the GIFT study. One individual carried the 3 variants and one individual carried the *SRBD1* variant only.

### Variants genotyped in individuals from GENES and MEGA

We examined the prevalence of the 6 rare candidate SNPs, *PLEKHH2* NM_172069:c.3105T>C, *LRPPRC* rs372371276, *SRBD1* rs34959371, *UNC5A* NM_133369.2:c.1869+23C>A, *GPRC5C* rs142232982 and, *RAB37* rs556450784, in 36 affected individuals from GENES and, 4,291 affected and 4,866 unaffected individuals from MEGA. We found additional carriers in MEGA, except for the *UNC5A* SNP. We initially found 2 carriers of the *PLEKHH2* variant (1 case and 1 control, and surprisingly, the case was homozygous for the minor allele) but after validation with Sanger sequencing, the case was found to be wild type. We also found 2 carriers of the *LRPPRC* variant (1 case and 1 control), 38 carriers of the *SRBD1* variant (14 cases and 24 controls), 19 carriers of the *GPRC5C* variant (7 cases and 12 controls), and 19 carriers of the *RAB37* variant (6 cases and 13 controls). Some individuals carried 2 of these disease candidate variants simultaneously: 1 control carried both *LRPPRC* and *SRBD1* variants, and 4 controls carried both *GPRC5C* and *RAB37* variants. We compared the distribution of the disease risk alleles between affected and unaffected individuals and found no statistically significant differences or clear signals of an association (Table 4).

**Table 4:** Candidate variants genotyped in MEGA.

Gene	Variant	Reference/alternate alleles	Number of MEGA cases			Number of MEGA controls			P-value	OR	95% CI
			Ref/Ref	Ref/Alt	Alt/Alt	Ref/Ref	Ref/Alt	Alt/Alt			
*PLEKHH2*	c.3105T>C	T/C	4031	0	0	4691	1	0	NA	NA	NA
*LRPPRC*	rs372371276	A/G	4044	1	0	4662	1	0	1	1.15	0.01–90.44
*SRBD1*	rs34959371	G/C	3829	14	0	4400	24	0	0.26	0.67	0.32–1.35
*UNC5A*	c.1869+23C>A	C/A	3981	0	0	4613	0	0	NA	NA	NA
*GPRC5C*	rs142232982	G/A	4021	7	0	4637	12	0	0.49	0.67	0.22–1.86
*RAB37*	rs556450784	G/A	4036	6	0	4598	13	0	0.25	0.53	0.16–1.48

### Association of SNPs from candidate genes with variability of hemostatic traits

We investigated whether any of the 5 candidate SNPs resulting from filter strategy 1 (*STX2* rs137928907, *ITGB3* rs5918, *APOH* rs4581, *KLK8* rs16988799, and *KLK11* rs3745539) were associated with one or more hemostatic traits known to associate with VTE. We investigated 413 individuals from GIFT, for whom both genotype and hemostatic traits data were available. No genotype was available or could be imputed for the *STX2* rs137928907. We found associations for the variants located in chromosome 17, *APOH* rs4581 and *ITGB3* rs5918. After correcting for multiple testing only one association remained significant. The *ITGB3* rs5918 remained associated with FX levels. All putative associations are shown in Table 5.

**Table 5:** Results of SNPs with P-values less than 0.05 for association with a haemostatic trait (samples from GIFT study). Putative associations were found for 2 of the candidate SNPs resulting from filter strategy 1. No genotype data was available for the *STX2* rs137928907. Abbreviations: NA, not available; -, no association; Ref, reference allele; Alt, alternate allele; N, number; CI, confidence interval.

Gene variant	Lab outcome	N individuals by genotype			Mean <lab outcome> by genotype			Multiple regression analysis				Single regression analysis
		Ref/Ref	Ref/Alt	Alt/Alt	Ref/Ref	Ref/Alt	Alt/Alt	Variance explained (%)	Per-Alt allele effect size ± Std. Error	P-value	95% CI	P-value
***STX2* rs137928907**	*NA*	*NA*	*NA*	*NA*	*NA*	*NA*	*NA*	*NA*	*NA*	*NA*	*NA*	*NA*
***ITGB3* rs5918**	Factor X levels	197	70	4	99 ± u/dL	107 ± 23 u/dL	114 ± 3 u/dL	6.7	9 ± 2 u/dL	3 × 10^−4^	3.91–13.19	0.001
	Factor IX levels	197	70	4	101 ± u/dL	106 ± u/dL	106 ± u/dL	5.6	5 ± 2 u/dL	0.013	1.14–9.68	0.029
	Protein S levels	197	70	4	93 ± u/dL	98 ± u/dL	101 ± u/dL	10.5	6 ± 2 u/dL	0.002	2.35–10.57	0.013
	Protein S free levels	197	70	4	88 ± u/dL	97 ± u/dL	88 ± u/dL	11.4	13 ± 3 u/dL	0.001	6.25–15.63	0.006
	Thrombin generation lagtime (Tissue factor = 1pM)	197	70	4	8.7 ±.1 min	9.7 ±.0 min	8.4 ±.4 min	8.2	0.9 ± 0.4 min	0.029	0.09–1.71	0.062
***APOH* rs4581**	Protein S free levels	182	78	11	88 ± u/dL	91 ± u/dL	106 ± u/dL	8.3	3 ± 3 u/dL	0.108	0.00–9.38	0.035
	Factor V ag	274	117	19	112 ± u/dL	104 ± u/dL	98 ± u/dL	2.6	-7 ± 3 u/dL	0.027	-12.49–-0.77	0.02
	Factor V act	274	116	18	103 ± u/dL	100 ± u/dL	95 ± u/dL	2.3	-3 ± 2 u/dL	0.037	-0.66–-0.21	0.032
	Thrombin generation lagtime (Tissue factor = 1pM)	182	78	11	8.7 ±.0 min	9.6 ±.0 min	10.0 ±.6 min	8	0.7 ± 0.4 min	0.04	0.03–1.44	0.032
	Thrombin generation lagtime (Tissue factor = 10pM)	182	78	11	1.9 ± 0.3 min	2.0 ± 0.4 min	2.0 ± 0.2 min	4.6	0.1 ± 0.0 min	0.018	0.02–0.17	0.014
***KLK8* rs16988799**	-	-	-	-	-	-	-	-	-	-	-	-
***KLK11* rs3745539**	-	-	-	-	-	-	-	-	-	-	-	-

We also investigated whether the 3 candidate variants that were identified from filter strategy 2, *RAB37* rs556450784, *GPRC5C* rs142232982 and *SRBD1* rs34959371, were associated with fibrinogen, total protein S and free protein S levels in MEGA (S4 File).

## Discussion

Our approach enabled us to narrow down the list of genetic variants to 3 candidate loci, but we were unable to identify the causative variant underlying the risk for disease in 2 families with unexplained familial VTE. Either variants in known hemostatic genes as well as rare variants across the exome are unlikely to explain the VTE tendency in these families.

A major strength of this study was the availability of families with multiple affected individuals that included cases with an early age of onset. Although family members are more likely to share environmental risk factors than unrelated individuals, young individuals without acquired causes for VTE are more likely to carry variants with large effect sizes [29]. Assuming that affected individuals from a family would all share the same susceptibility allele, we could reduce the list of candidate SNPs from 121,347 to 1,238, and candidate INDELs from 23,364 to 163 before applying more stringent filter strategies. It is worth mentioning that an analysis-by-exclusion approach with exomes from unaffected relatives could have reduced the number of shared variants to a minimum. We chose to only sequence exomes from affected relatives because an analysis-by-exclusion also has its limitations in VTE family studies, as we could have missed disease risk variants because unaffected individuals from the same family can still develop the disease at older age.

Our choice to pursue a high-throughput exome sequencing approach enabled us to identify both rare and common coding variants. Moreover, this approach allowed us to focus not only on variants located in genes previously reported to be associated with VTE, but also in novel genes.

Many patients and families included in gene identification studies still remain without a molecular diagnosis [30]. One of the main reasons is because ranking variants is often a challenge in these studies [31]. We reported our results from applying three different filter strategies, as reporting the results from different approaches is essential to improve variant ranking strategies. One common approach consists of prioritizing non-synonymous variants located in or near genes known to be associated with the disease of interest or a related phenotype. These variants are much more likely to be involved in the disease than variants elsewhere in the genome. This is the foundation of our filter strategy 1. Another approach consists of prioritizing rare and low frequency variants. Rare and low frequency variants are often at the basis of inherited risk for VTE, like in the case of deficiencies in one of the natural anticoagulants and the factor V Leiden and prothrombin mutations [30]. The use of rare and low frequency variants is the foundation of the remaining two filter strategies. Compared with the filter strategy 2, the filter strategy 3 has a more relaxed MAF threshold since we cannot exclude that young controls included in our study might develop VTE associated with inherited thrombophilia at a later time. Data from a set of cases with family history of VTE (GIFT) was key to rank these variants although we cannot ignore the possibility of generating false negative results due to the small sample size (only 96 unrelated cases). Of note, since there is growing evidence that supports a functional role for synonymous rare codons [32,33], we retained synonymous variants for analysis in both filter strategies 2 and 3.

Several genome-wide studies, and more recently also whole exome sequencing studies have been conducted to identify genetic risk factors for VTE [9–14,34–36]. However, nearly all variants that have been robustly found to be associated with the risk for this disease are located within known susceptibility genes. In our study, the candidate variants resulting from filter strategy 1 are the least likely disease-causing variants, as there were a high number of unaffected carriers, including homozygotes. Furthermore, because the affected individuals from these families do not share abnormalities in hemostatic traits known to affect the risk of VTE, variants with a large effect size are unlikely to be present among the known VTE susceptibility genes.

All variants tested for segregation with the disease showed incomplete penetrance, i.e. unaffected individuals carrying the putative pathogenic variant were found among the families. Yet, an autosomal dominant incomplete penetrance is likely. Despite the inheritance pattern being consistent with an autosomal dominant mode for both families, family K oldest generation (proband’s parents) did not experience VTE. Furthermore, given the different clinical manifestations of VTE within the affected individuals, it is plausible that other factors, such as environmental or other genetic modifiers, are also involved in the pathogenesis of the disease in these families. Although some studies suggest that the proportion of the variance attributable to shared familial environment factors is small [37,38], it is difficult to predict the likelihood of finding VTE-causing variants based solely on the severity of the disease within a single family.

With regard to the 6 variants genotyped in MEGA, we found additional carriers among cases and controls, except for the UNC5A variant, for which no carrier was found. The 2 putative pathogenic variants identified for family D, the *GPRC5C* and *RAB37* SNPs, were found to be more prevalent in the Netherlands than in the worldwide population. This was unexpected because none of these variants was present in GoNL database. An explanation could be that these genomic regions were not covered in GoNL database. With regard to the 4 putative pathogenic variants identified in family K, the rarity of the variants identified in *UNC5A*, *LRPPRC* and *PLEKHH2* genes suggests that these variants are deleterious. However, whether these variants predispose to VTE remains unknown. The *UNC5A* variant seems to be a family-specific variant, as we have not identified additional carriers nor it is reported in any of the available database. The *PLEKHH2* variant was found in 1 unaffected individual while the *LRPPRC* variant was found 1 affected and 1 unaffected individual, both with negative family history for VTE. Noteworthy that despite being so rare, both *LRPPRC* and *PLEKHH2* variants were found in 1 affected individual with positive family history for VTE in GIFT. Co-segregation with the phenotype in other affected family members may shed further light on the causality of this locus. This is particularly important because these variants are predicted to have no functional consequence and biological evidence might be difficult to address.

According to the GWAS catalog database, variants in *SRBD1*, *LRPPRC* and *RAB37* genes have been reported to be associated with other phenotypes: *SRBD1* rs3213787 has been associated with normal tension glaucoma[39], *LRPPRC* rs13387221 has been associated with cognitive ability (intelligence) in childhood[40] and *RAB37* rs10512597 has been associated with fibrinogen levels[41,42]. This last association is intriguing because elevated total fibrinogen levels and reduced fibrinogen gamma′ levels are associated with increased risk for VTE[43]. Common SNPs at the locus 17q25.1, which includes the *RAB37* rs10512597-C, have been associated with reduced fibrinogen and C-reactive protein levels[41,42], which suggests that regulators of fibrinogen and C-reactive protein levels might be present at this locus. However, in the GIFT study, the *RAB37* rs10512597-C was not associated with fibrinogen levels (β = 0.07, 95% CI = -0.05–0.20, p = 0.251) but instead, it was associated with protein S levels (β = 4.37, 95% CI = 1.00–7.74, p = 0.011) but not free protein S levels (β = 0.010, 95% CI = -0.006–0.026, p = 0. 236) (MLRC, PHR, unpublished observation August 2015). Both candidate variants from family D, *RAB37* rs556450784 and *GPRC5C* rs142232982, were not associated with fibrinogen or protein S levels in MEGA.

Variants located within the nearest upstream or the nearest downstream genes of the following candidate genes, *PLEKHH2*, *LRPPRC*, and *SRBD2*, all located in the 2p21 locus, have also been reported as associated with various phenotypes according to the GWAS catalog database. Variants in *THADA* have been associated with age-related hearing impairment[44], Crohn's disease[45], DNA methylation variation[46], hair morphology[47], inflammatory bowel disease[48], mitochondrial DNA levels[49], orofacial clefts[50,51], platelet counts[52], polycystic ovary syndrome[53,54], prostate cancer[55], response to amphetamines[56], and type 2 diabetes[57]. Variants in *ABCG8* have been associated with LDL cholesterol[58–63], total cholesterol[58–60], campesterol levels[64], and gallstones[65]. Finally, variants in *PRKCE* have been associated with various red blood cell traits (red blood cell counts[66], hematocrit[66–69], and hemoglobin[66,67]), as well as pulmonary function decline[70], QT interval[71], metabolite levels (X-11787)[72], and suicide risk[72]. While some of these phenotypes might be linked with the risk of developing VTE, the association of the 2p21 locus with VTE risk has not been reported before.

Our study has some limitations. First, although different types of genetic variation exist, we have investigated only SNPs and INDELs. Second, we had to employ different filtering strategies to select only potentially clinically relevant variants, as the number of variants shared by the affected family members was very high. We cannot exclude the possibility of missing the causative variant after applying these strategies. Nevertheless, because there are no established filtering strategies for identification of VTE causing genes, we have validated with Sanger sequencing additional variants that were not retained after applying the different filter strategies (details in S2 File). Yet, none of these additional variants is likely to explain the increased risk for VTE in families D and K. As a side note, researchers can reutilize these data and apply filter strategies other than the ones described in our manuscript to identify putative disease-causing variants (the information about all variants shared by the relatives can be found in S2 File). For example, researchers can prioritize the variants based on other candidate genes [73] or on scores that estimate the variant effect [74] and compare these with their relevant datasets. Third, given that all 10 samples from the 2 families have undergone the same experimental protocol and bioinformatics analysis, systematic errors are likely to be present in this dataset. Some of these errors might lead to erroneous variant calls. To minimize this type of errors, for each variant identified in our analysis, we have obtained the genotype information for all samples. This allowed us to distinguish samples with homozygous reference calls from samples with no genotype calls. We used this information and selected only SNPs for which the genotype information was available in all 10 individuals. Therefore, although discerning rare variants from sequencing errors remains a big challenge in next generation sequencing data analysis, we think that rare variants exclusively shared by one family are less likely to be sequencing errors in our study. Yet, we cannot exclude the possibility of incomplete coverage of some genes, a limitation inherent to the next generation sequencing technique. Fourth, although all variants analyzed are based on families D and K results, the GIFT, EVS_EA and GoNL data were generated with different library preparation, sequencing and bioinformatics methodology. Cross-dataset comparisons (comparing GIFT cases with EVS_EA or GoNL) are in general prone to technical bias. Fifth, our sample size in MEGA was powered to detect genotype relative risks greater than 4 at the significance level of 0.05 for risk allele frequencies greater than 0.10%, and the power to detect a relative risk of 1.5 was low. Sixth, although our results argue that coding variants in known genes or rare coding variants across the exome are unlikely to explain the VTE tendency in these families, these results are not generalizable to other families with unexplained VTE. Finally, our whole exome sequencing data was generated by the end of 2011 and a significant improvement on sequencing technology and data analysis has been made over the last years. The feasibility of whole exome sequencing for identifying new VTE genetic variants is only as good as the quality of the data, so it could be that better coverage might lead to identification of more potential variants of interest.

In conclusion, despite extensive investigation, we did not find a definitive genetic cause for the increased risk of VTE in the 2 evaluated families. Our study suggests that rare variants within 3 candidate loci, 2p21, 5q35.2 and 17q25.1, have an impact on the risk of VTE in these families, but we could not find any evidence for the 6 rare variants genotyped in a large case-control association study.

## Supporting information

S1 File List of 126 genes. An excel workbook with information concerning the genes names and genomic location.
(XLSX)

S2 File Variants shared by all affected members. An excel workbook that includes the following information: all variants retained for manual inspection after applying the 3 filter strategies, all variants shared by family members with Filter = PASS, and (iii) all variants shared by family members with Filter = NO PASS. All variants validated with Sanger sequencing are discernible. Please note that some of these variants did not follow our filter strategies described in the manuscript.
(XLSX)

S3 File Results of Sanger sequencing in affected and unaffected family members for all candidate variants. (XLSX)

S4 File Association analysis of fibrinogen, total and free protein S levels in healthy individuals from MEGA. (DOCX)
