# Supplementary material for: Whole exome sequencing in thrombophilic pedigrees to identify genetic risk factors for venous thromboembolism
Source: PLoS One. 2017 Nov 8;12(11):e0187699. doi: 10.1371/journal.pone.0187699 (PMC5695603; doi:10.1371/journal.pone.0187699)
Supplement: S4 File — (DOCX) [file pone.0187699.s004.docx]

Table S1 Association analysis of fibrinogen, total and free protein S levels in healthy individuals from MEGA.

| Variant | Genotype | Controls heterozygous N (N with levels) | Fibrinogen Mean (g/L) | Mean difference (95% CI) | Protein S Mean (U/dL) | Mean difference (95% CI) | Free protein S Mean (U/dL) | Mean difference (95% CI) |
| --- | --- | --- | --- | --- | --- | --- | --- | --- |
|  |  |  |  |  |  |  |  |  |
| *RAB37* rs556450784 | GA | 13 (8) | 3.19 | 0.13 (-0.34 to 0.59) | 93.8 | 8.19 (-5.78 to 22.17) | 86.4 | 5.2 (-11.2 to 21.7) |
|  | GG | 4598 (2906) | 3.31 |  | 102.0 |  | 91.6 |  |
| *GPRC5C* rs142232982 | AG | 12 (6) | 3.08 | 0.24 (-0.30 to 0.77) | 99.1 | 2.95 (-13.2 to 19.1) | 92.1 | -0.5 (-18.2 to 17.3) |
|  | GG | 4637 (2905) | 3.31 |  | 102.0 |  | 91.6 |  |
| *SRBD1* rs34959371 | CG | 24 (17) | 3.27 | 0.05 (-0.27 to 0.37) | 100.4 | 1.59 (-8.00 to 11.17) | 85.9 | 5.8 (-4.7 to 16.4) |
|  | GG | 4400 (2888) | 3.31 |  | 102.0 |  | 91.7 |  |
